# Biglycan is a specific marker and an autocrine angiogenic factor of tumour endothelial cells

**DOI:** 10.1038/bjc.2012.59

**Published:** 2012-02-28

**Authors:** K Yamamoto, N Ohga, Y Hida, N Maishi, T Kawamoto, K Kitayama, K Akiyama, T Osawa, M Kondoh, K Matsuda, Y Onodera, M Fujie, K Kaga, S Hirano, N Shinohara, M Shindoh, K Hida

**Affiliations:** 1 Department of Vascular Biology, Graduate School of Dental Medicine, N13 W7, University of Hokkaido, Sapporo, Hokkaido 060-8586, Japan; 2 Department of Gastroenterological Surgery II, Graduate School of Medicine, N15 W7, University of Hokkaido, Sapporo, Hokkaido 060-8638, Japan; 3 Department of Cardiovascular and Thoracic Surgery, Graduate School of Medicine, N15 W7, University of Hokkaido, Sapporo, Hokkaido 060-8638, Japan; 4 Department of Oral Pathology and Biology, Graduate School of Dental Medicine, N13 W7, University of Hokkaido, Sapporo, Hokkaido 060-8586, Japan; 5 DNA Sequencing Center Section, Okinawa Institute of Science and Technology Promotion Corporation, Onna, Okinawa 904-0412, Japan; 6 Department of Renal and Genitourinary Surgery, Graduate School of Medicine, N15 W7, University of Hokkaido, Sapporo, Hokkaido 060-8638, Japan

**Keywords:** biglycan, tumour endothelial cells, tumour angiogenesis

## Abstract

**Background:**

We isolated tumour endothelial cells (TECs), demonstrated their abnormalities, compared gene expression profiles of TECs and normal endothelial cells (NECs) by microarray analysis and identified several genes upregulated in TECs. We focused on the gene encoding biglycan, a small leucine-rich repeat proteoglycan. No report is available on biglycan expression or function in TECs.

**Methods:**

The NEC and TEC were isolated. We investigated the biglycan expression and function in TECs. Western blotting analysis of biglycan was performed on sera from cancer patients.

**Results:**

Biglycan expression levels were higher in TECs than in NECs. Biglycan knockdown inhibited cell migration and caused morphological changes in TECs. Furthermore, immunostaining revealed strong biglycan expression *in vivo* in human tumour vessels, as in mouse TECs. Biglycan was detected in the sera of cancer patients but was hardly detected in those of healthy volunteers.

**Conclusion:**

These findings suggested that biglycan is a novel TEC marker and a target for anti-angiogenic therapy.

Tumour blood vessels have been recognised as an important target for cancer therapy after (Folkman, 1971) proposed that tumour growth depends on angiogenesis. Growing tumours produce growth factors and cytokines that are responsible for the remodelling of the pre-existing vascular network by angiogenic sprouting and neovascularisation (Bergers and Benjamin, 2003; Wang *et al*, 2010). In addition, these vessels function as gatekeepers for tumour cells to metastasise to other organs (Folkman, 2002). Therefore, inhibiting tumour angiogenesis is a promising strategy for cancer treatment.

Tumour blood vessels differ from their normal counterparts in several ways, such as changes in morphology, altered blood flow and enhanced leakiness (McDonald and Baluk, 2002; Morikawa *et al*, 2002; Jain, 2003). In addition, gene expression profiles of tumour endothelial cells (TECs) differ from those of normal endothelial cells (NECs) (St Croix *et al*, 2000; McDonald and Baluk, 2002; Morikawa *et al*, 2002). The TECs grow faster and migrate better than NECs (Matsuda *et al*, 2010).

The TECs are more sensitive to certain drugs, such as cyclooxygenase-2 inhibitors and the polyphenol epigallocatechin-3 gallate in green tea (Ohga *et al*, 2009; Muraki *et al*, 2012). Furthermore, TECs are cytogenetically abnormal (Hida *et al*, 2004; Akino *et al*, 2009).

The currently used anti-angiogenic therapies have been reported to cause side effects such as haemoptysis and intestinal perforation (Johnson *et al*, 2004; Kindler *et al*, 2005; Keedy and Sandler, 2007; Saif *et al*, 2007). Many of these therapies block important angiogenic factors or their signalling, including vascular endothelial growth factor (VEGF), which are required for the maintenance of normal endothelium. These therapies occasionally damage NECs. To develop a novel target for anti-angiogenic therapy that is specific for TECs, we performed DNA microarray analysis and found that biglycan was upregulated more than about 100-fold in TECs compared with NECs.

Biglycan belongs to the family of small leucine-rich proteoglycans and consists of a core protein of 331 amino acids covalently bound to two chondroitin sulphate- or dermatan sulphate-containing glycosaminoglycan side chains (Bianco *et al*, 1990). Biglycan is strongly expressed in inflammatory and fibrotic tissue (Westermann *et al*, 2008; Babelova *et al*, 2009; Mohan *et al*, 2010), and it induces cytoskeletal changes in lung fibroblast that results in increased cell migration (Tufvesson and Westergren-Thorsson, 2003).

A recent study has shown that the proteoglycans contribute to tumour progression (Yang *et al*, 2007). However, there are few reports on biglycan expression and function in the tumour microenvironment.

In this study, we investigated biglycan expression and function in tumour blood vessels and addressed the possibility that it can be a novel TEC marker.

## Materials and Methods

### Chemicals

Biglycan was purchased from Sigma Chemical Co (St Louis, MO, USA). Fluorescein Isothiocyanate (FITC)-conjugated lectin *Bandeiraea simplicifolia* isolectin B4 (BS1-B4) was purchased from Vector Laboratories (Burlingame, CA, USA).

### Cell line and culture conditions

Super-metastatic human melanoma cells (A375SM cells), kindly gifted by Dr Isaiah J Fidler (MD Anderson Cancer Center, Houston, TX, USA), were cultured as described previously (Ohga *et al*, 2012).

### Antibodies

The following antibodies were used: goat anti-mouse biglycan from Abcam (Cambridge, UK); mouse anti-human vinculin antibody (Sigma Chemical Co.); rat anti-mouse CD31 antibody from eBioscience (San Diego, CA, USA) and FITC-anti-mouse CD31 antibody (eBioscience); PE-anti-mouse CD31 antibody from BD Pharmingen (San Diego, CA, USA), anti-mouse CD105 antibody (BD Pharmingen) and rat anti-mouse CD144 antibody (BD Pharmingen); Alexa Fluor 594 goat anti-rat IgG antibody from Invitrogen (Tokyo, Japan), Alexa Fluor 488 donkey anti-goat IgG antibody (Invitrogen) and Alexa Fluor 594 goat anti-rabbit IgG antibody (Invitrogen) and monoclonal anti-*β*-actin (AC-15) antibody from Sigma-Aldrich (St Louis, MO, USA).

### Isolation of TECs and NECs

As described previously, TECs were isolated from human tumour xenografts (melanoma) in nude mice and NECs (skin) were isolated from the dermis of the nude mice as controls (Hida *et al*, 2004; Kurosu *et al*, 2011; Akiyama *et al*, 2012; Ohga *et al*, 2012). All procedures for animal experiments were approved by the local animal research authorities, and animal care was performed in accordance with institutional guidelines.

### Flow cytometry

The cells were analysed on a FACSAria II obtained from Becton Dickinson (San Jose, CA, USA), using the FITC-conjugated BS1-B4 lectin and antibodies against CD31, CD105 and CD144. Representative data were analysed using FlowJo software obtained from Treestar (Ashland, OR, USA).

### Microrray gene expression analysis

Total RNA was isolated from three types of TECs (melanoma-derived ECs, renal carcinoma-derived ECs and oral carcinoma-derived ECs) and NECs with TRIzol (Invitrogen), according to the manufacturer's standard protocol. The quality of RNA was tested by electrophoresis using an Agilent 2100 Bioanalyzer (Agilent Technologies, Santa Clara, CA, USA). Total RNA was labeled with Cyanin-5 CTP by linear amplification using a Low RNA Input Fluorescent Linear Amplification Kit (Agilent Technologies) as specified by the manufacturer. The quality and size distribution of labeled cRNA were determined by 2100 Bioanalyzer (Agilent Technologies) and quantified using a NanoDrop microscale spectrophotometer purchased from NanoDrop Technologies (Rockland, DE, USA). A set of 5 *μ*g fluorescent-labeled cRNA targets from each sample was assembled into a hybridisation reaction on the Mouse Oligo Microarray (Agilent Technologies) using the *In Situ* Hybridization Kit Plus (Agilent Technologies). Washing, signal scanning, image analysis and data extraction was performed as described previously (Ishibashi *et al*, 2005).

### Reverse transcription–PCR (RT–PCR) and quantitative real-time RT—PCR

Total RNA was extracted and first-strand complementary DNA was synthesised using the RNeasy Micro Kit obtained from Qiagen (Valencia, CA, USA) from each EC type. Real-time RT–PCR was performed as described previously (Kurosu *et al*, 2011). The primers used for RT–PCR are indicated in Supplementary Figure S1.

### Tube formation assay

ECs were seeded at a density of 1 × 10^5^ cells per well and incubated at 37°C on Matrigel (BD Biosciences, San Jose, CA, USA) as described previously (Kurosu *et al*, 2011). Tube formation was observed using an inverted microscope by measuring the length of tubes. For inhibition experiments, TECs were preincubated for 8 h at 37°C with anti-TLR2, anti-TLR4 blocking (BioLegend, San Diego, CA, USA) and isotype control antibodies (IgG2a; BioLegend).

### Western blotting

Western blotting was performed using antibodies specific to biglycan and *β*-actin, and an HRP-conjugated secondary antibody as described previously (Ohga *et al*, 2009; Kurosu *et al*, 2011). The level of biglycan was normalised to that of *β*-actin by scanning densitometry using Image J software from NIH (Bethesda, MD, USA). Experiments were performed three times.

### Immunocytochemistry and immunohistochemistry

Tumour endothelial cells and NECs were fixed in cold methanol and immunostained with the anti-biglycan antibody and then with the secondary antibody. Mouse tumour tissues were dissected from killed mice. Human tissue samples were obtained at Hokkaido University Hospital. Informed consent was obtained from all patients before the samples were used. Frozen sections were prepared as described previously, (Ohga *et al*, 2012) and were double stained using anti-CD31 and anti-biglycan to show the colocalisation of CD31 and biglycan in ECs. All immunostained samples were counterstained with DAPI (Roche Diagnostics, Mannheim, Germany) and visualised using an Olympus FluoView FV1000 confocal microscope (Olympus, Tokyo, Japan).

### Biglycan knockdown

Biglycan siRNA was transfected using Lipofectamine transfection reagent (Invitrogen) according to the manufacturer's instructions. The sequence of the biglycan siRNA was 5′-AAACCCUUCUGCUCAAAGGGCAAGG-3′, and the control siRNA was a non-targeting control (Qiagen).

### Cell migration assay

Cell migration towards VEGF was analysed using a Boyden chamber (Neuro Probe Inc., Gaithersburg, MD, USA), as previously described with modifications (Ohga *et al*, 2009; Muraki *et al*, 2012). Vascular endothelial growth factor-A (10 ng ml^−1^) was added to the lower chambers as a chemoattractant. TECs were treated with the control siRNA (10 *μ*M) or biglycan siRNA (10 *μ*M) in EGM-2MV for 72 h. In total, 1.5 × 10^4^ cells were seeded in the upper chambers and incubated for 4 h at 37°C.

### Cell proliferation assay

The TECs were treated with control siRNA (10 *μ*M) and biglycan siRNA (10 *μ*M). After siRNA transfection for 24 h, 1 × 10^3^ cells per well were seeded into 96 dishes in EBM-2 with 0.5% FBS. Cell proliferation was measured every day for 3 days by MTS assay.

### Supernatant condensation

The supernatants were collected and concentrated ∼ 120-fold using Amicon Ultra-15 30K centrifugal filter units obtained from Millipore (Billerica, MA, USA) and Amicon Ultra 0.5 ml 30K centrifugal filters (Millipore). The concentrates were analysed by western blotting.

### Glycoprotein extraction in human serum

Glycoproteins in human serum were enriched using Glycoprotein Enrichment Resin (Clontech, Palo Alto, CA, USA), and biglycan expression was analysed by western blotting.

### Statistical analysis

Differences between experimental groups were evaluated using the Mann–Whitney *U*-test. *P*<0.05 was considered significant, and *P*<0.01 was considered highly significant.

## Results

### Isolation and characterisation of TECs and NECs

The TECs were isolated from A375SM xenografts in nude mice, and NECs (skin ECs) were isolated from the dermal tissue of the nude mice (Hida *et al*, 2004).

**Figure 1:**
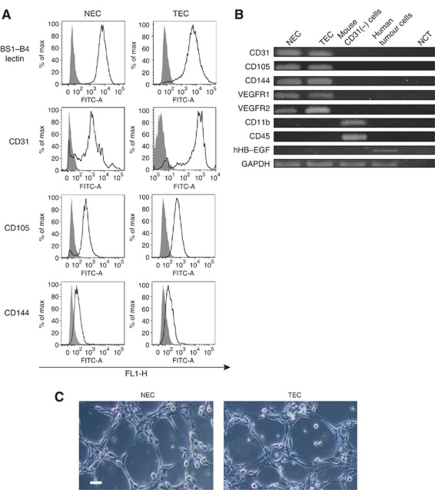
Characterisation of isolated TECs and NECs. (**A**) The binding of lectin BS1-B4 and expression of CD31, CD105 and CD144 (blue line) indicated the high purity of isolated TECs and NECs. The isotype control is shown as a red line. (**B**) Cultured TECs and NECs were positive for CD31, CD105, CD144, VEGFR-1 and VEGFR-2 by RT–PCR. Mouse tumour stromal CD31 (−) cells were also included in the samples. TECs and NECs were negative for the monocyte marker CD11b and haematopoietic marker CD45. Human HB–EGF expression was detected in human tumour cells but not in TECs or NECs. Abbreviations: NCT=negative control template. (**C**) Isolated and cultured ECs formed tubes on matrigel-coated plates. Scale bar, 10 *μ*m.

According to flow cytometric analysis, the binding of lectin BS1-B4 and expression of CD31, CD105 and CD144 indicated the high purity of isolated ECs (Figure 1A). Furthermore, RT–PCR revealed expression of the following endothelial markers in TECs and NECs: CD31, CD105, CD144, VEGFR-1 and VEGFR-2 (Figure 1B). Isolated ECs were negative for the monocyte marker CD11b and haematopoietic marker CD45. Human cells expressed human HB–EGF. No human HB–EGF mRNA expression was detected in mouse TECs, demonstrating that these TECs were not contaminated with human tumour cells. These results excluded the possibility that isolated and cultured ECs were contaminated with non-ECs.

In addition, cultured ECs formed tubes on Matrigel-coated plates (BD Biosciences) (Figure 1C). The cultured TECs and NECs maintained the properties of EC after isolation.

### Biglycan is specifically expressed in TECs

The DNA microarray showed that about 70 genes were upregulated in TECs compared with NECs. Biglycan mRNA expression level was >100-fold higher in TECs than in NECs (Supplementary Figure S2).

**Figure 2:**
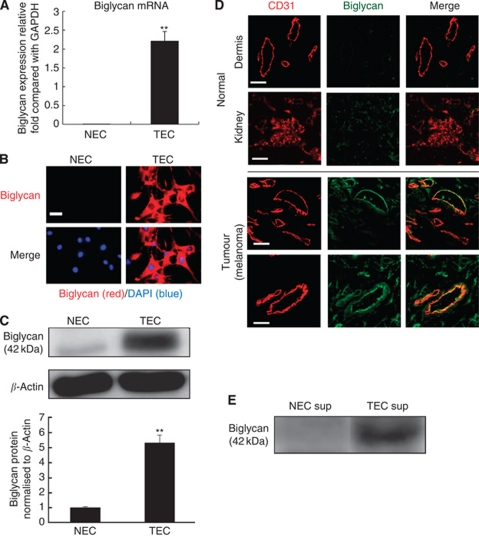
Biglycan is specifically expressed in mouse TECs. (**A**) The relative expression of biglycan to that of GAPDH in TECs and NECs was measured using quantitative real-time RT–PCR (^**^P<0.01). (**B**) Biglycan protein expression was analysed by fluorescent immunocytochemistry. Biglycan was detected in TECs but not in NECs. Blue: DAPI, Red: biglycan. Scale bar, 25 *μ*m. (**C**) Western blotting revealed that biglycan protein expression was upregulated in TECs than in NECs. Representative data are shown from one of three experiments. The level of biglycan was normalised to that of *β*-actin and was analysed by scanning densitometry using Image J software from NIH (^**^P<0.01). (**D**) Biglycan expression in tumour tissues dissected from mice xenografted human tumour cells (A375SM), normal dermal tissue and normal kidney tissue. Fluorescent immunohistochemical staining with the biglycan antibody revealed biglycan (green stain) predominantly in the tumour vessels of mice xenografted human tumour cells. Scale bar, 50 *μ*m. (**E**) Western blotting revealed that biglycan expression was detected in the TEC supernatant but hardly detected in the NEC supernatant. Representative data are shown from one of three experiments.

Real-time PCR revealed that biglycan mRNA expression was upregulated in TEC than in NEC (Figure 2A). Immunocytochemical staining revealed that biglycan was expressed in TEC but not in NEC (Figure 2B). As biglycan is very similar to other proteoglycans, we checked the specificity of RT–PCR for biglycan. Any other proteoglycans were not amplified by the RT–PCR protocol (Supplementary Figure S3). PCR product for human decorin by biglycan primer should be theoretically 779 bp; however, this size of product was not detected.

In addition, western blotting revealed that the biglycan protein was specifically expressed in TEC (Figure 2C). These findings suggested that biglycan expression was upregulated in TEC at both the mRNA and protein levels.

To analyse biglycan expression in tumour blood vessels *in vivo*, immunofluorescent double staining was performed using cryosections of the human tumour xenografts, from which TECs were isolated, and mouse normal dermis and kidney tissues. Immunostaining clarified the colocalisation of CD31 and biglycan. Biglycan was stained in tumour blood vessels, but not or weakly stained in normal blood vessels (Figure 2D).

To explore whether biglycan is secreted by TECs, EC supernatants were collected and analysed by western blotting. The biglycan protein was detected in the TEC supernatant, but was hardly detected in the NEC supernatant (Figure 2E).

These results suggested that biglycan is specifically expressed in mouse TECs *in vitro* and *in vivo*.

### Biglycan knockdown inhibits TEC migration and tube formation

**Figure 3:**
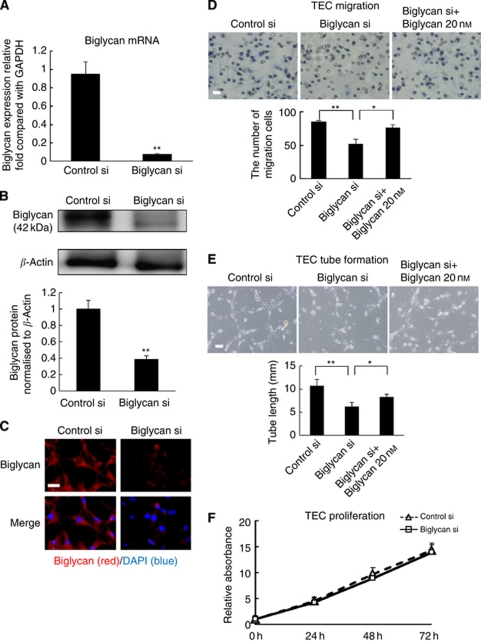
Biglycan knockdown inhibited TEC migration and tube formation. (**A**) Silencing of biglycan mRNA was confirmed by quantitative real-time RT–PCR (^**^P<0.01). (**B** and **C**) Silencing of the biglycan protein was confirmed by immunocytochemistry and western blotting (^**^P<0.01). Scale bar, 25 *μ*m. (**D**) Migration towards VEGF was significantly inhibited by biglycan siRNA in TECs (^**^P<0.01). When biglycan knockdown TECs were treated with exogenous biglycan protein, migration towards VEGF was restored (^*^P<0.05). Scale bar, 100 *μ*m. (**E**) Tube formation was significantly inhibited by biglycan knockdown (^**^P<0.01). Exogenous biglycan protein restored the length of tube in biglycan knockdown TECs (^*^P<0.05). Representative figures are shown. Scale bar, 100 *μ*m). (**F**) TEC proliferation was not affected by biglycan siRNA.

To analyse the role of biglycan in TECs, TECs were subjected to RNAi using biglycan siRNA to silence biglycan mRNA expression. Biglycan knockdown in TECs was confirmed at the mRNA level by real-time PCR (Figure 3A), and at the protein level by western blotting and densitometry analysis (Figure 3B). In addition, immunocytochemistry confirmed biglycan knockdown in TECs (Figure 3C).

To analyse the effects of biglycan knockdown on the proangiogenic properties of TECs, cell migration towards VEGF was analysed using the Boyden chamber. Biglycan knockdown significantly inhibited TEC migration towards VEGF (Figure 3D). The number of migrated cells was restored by treatment with the biglycan protein (20 nM) (Figure 3D). Biglycan enhanced cell migration in a dose-dependent manner (Supplementary Figure S4). The data suggested that biglycan promotes the migration of TECs.

To analyse another effect of biglycan knockdown on angiogenic properties of TECs, we performed tube formation assay. Representative data and a quantitative analysis of tube length are shown (Figure 3E). The ability to form capillary-like structures was impaired by biglycan knockdown in TEC. The tube formation in TEC was restored by treatment with biglycan, suggesting that biglycan has an important role for TEC tube formation. However, biglycan knockdown did not influence cell proliferation (Figure 3F).

### Biglycan knockdown caused morphological changes in TECs

**Figure 4:**
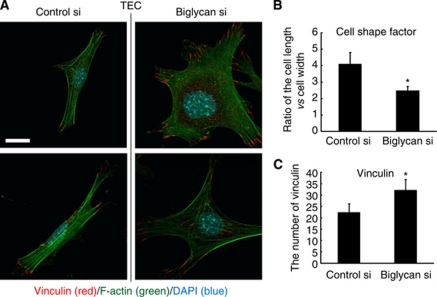
Biglycan knockdown caused morphological changes in TECs. (**A**) TECs with biglycan knockdown became more spread. Vinculin expression increased in TECs with biglycan knockdown. Scale bar, 10 *μ*m. (**B**) The ratio of cell length *vs* cell width was (^*^P<0.05) decreased in TECs with biglycan knockdown. (**C**) Vinculin expression was increased in TECs with biglycan knockdown (^*^P<0.05).

The cell morphology and cytoskeleton are involved in cell migration (Small, 1981). To analyse the morphological changes in TECs with biglycan knockdown, the cells were stained with anti-phalloidin antibody that reveals F-actin. After biglycan mRNA expression was silenced in TECs, their shape became more spread (Figure 4A). The ratio of cell length to cell width was lower in TECs with biglycan knockdown than in control TECs (Figure 4B).

Cell migration is coordinated by a complex of proteins that localises to the sites of the cell–matrix interaction, the focal adhesions (Humphries *et al*, 2007). The adaptor protein vinculin is a key regulator of focal adhesions and its overexpression suppresses cell migration (Coll *et al*, 1995). Biglycan knockdown resulted in increased vinculin expression in TECs (Figures 4A and C). These results suggested that biglycan contributes to cell morphology and cell migration in TECs.

### Biglycan activated NEC migration and tube formation

**Figure 5:**
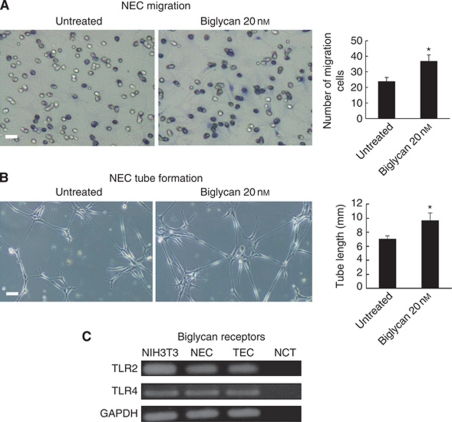
Biglycan activated NEC migration and tube formation and ECs expressed biglycan receptors. (**A**) When NECs were treated with exogenous biglycan (20 nM), the number of cells migrating towards VEGF increased (^*^P<0.05). Scale bar, 100 *μ*m. (**B**) When NECs were treated with exogenous biglycan (20 nM), the length of tube increased (^*^P<0.05). Scale bar, 100 *μ*m. (**C**) Expressions of biglycan receptors, TLR2 and TLR4, were analysed in TECs by RT–PCR. Both receptors were expressed in in TECs and NECs. Abbreviation: NCT=negative control template.

To analyse the involvement of biglycan for acquisition of angiogenic phenotypes in TEC, NECs were treated with biglycan (20 nM) for 24 h. The number of migrated cells and tube length in NEC increased by treatment with biglycan protein (Figures 5A and B). These data suggested that biglycan induces the angiogenic phenotypes in NEC.

### Biglycan acts in an autocrine manner in TEC through TLR2 and TLR4

Because biglycan protein reversed the biglycan knockdown-mediated suppression of cell migration and tube formation, it was speculated that ECs express biglycan receptors. TLR2 and TLR4 are the receptors of biglycan (Schaefer *et al*, 2005). The expression of TLR2 and TLR4 was detected in TECs by RT–PCR (Figure 5C).

**Figure 6:**
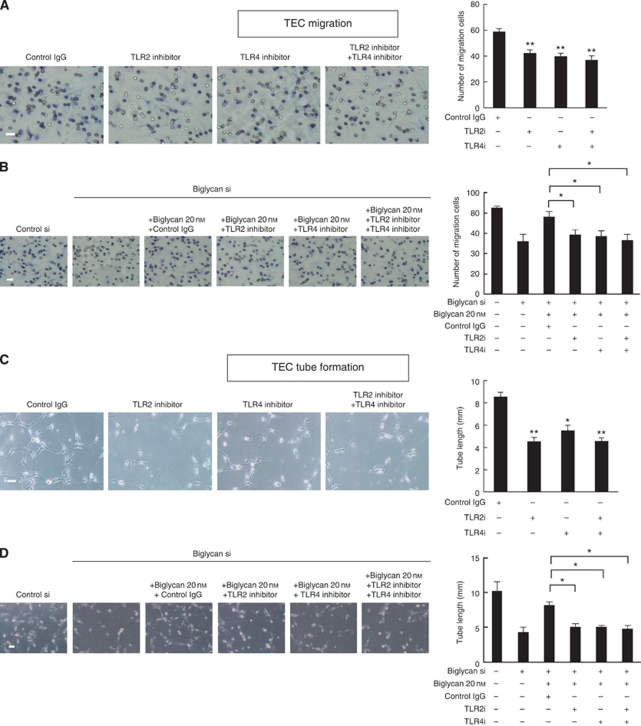
Biglycan acts in an autocrine manner through TLR2 and TLR4. (**A**) TECs migration was inhibited by anti-TLR2 or anti-TLR4 antibodies (^**^P<0.01). Scale bar, 100 *μ*m. (**B**) In biglycan knockdown TECs, the biglycan-induced cell migration was inhibited by blocking anti-TLR2 or anti-TLR4 antibodies (^*^P<0.05). Scale bar, 100 *μ*m (**C**) TECs tube formation was inhibited in the presence of blocking anti-TLR2 or anti-TLR4 antibodies (^**^P<0.01, ^*^P<0.05). Scale bar, 100 *μ*m. (**D**) In biglycan knockdown TECs, the biglycan-induced tube formation was suppressed by blocking anti-TLR2 or anti-TLR4 antibodies (^*^P<0.05). Scale bar, 100 *μ*m.

To analyse the role of TLR2 and TLR4 in proangiogenic responses of biglycan, we tested the anti-TLR2 and anti-TLR4 antibodies. TLR2 and TLR4 antibodies suppressed TEC migration and tube formation (Figures 6A and C). Furthermore, the biglycan-induced migration and tube formation was cancelled by anti-TLR2 and anti-TLR4 antibodies in biglycan knockdown TEC (Figures 6B and D) and NEC (Supplementary Figure S5). These results suggested that biglycan acts in an autocrine manner in TEC through TLR2 and TLR4.

### Human TECs expressed higher levels of biglycan *in vitro* and *in vivo*

To analyse biglycan expression in human TECs and NECs, we isolated TECs from human renal cell carcinoma tissue and NECs from normal renal tissue in the same patients.

**Figure 7:**
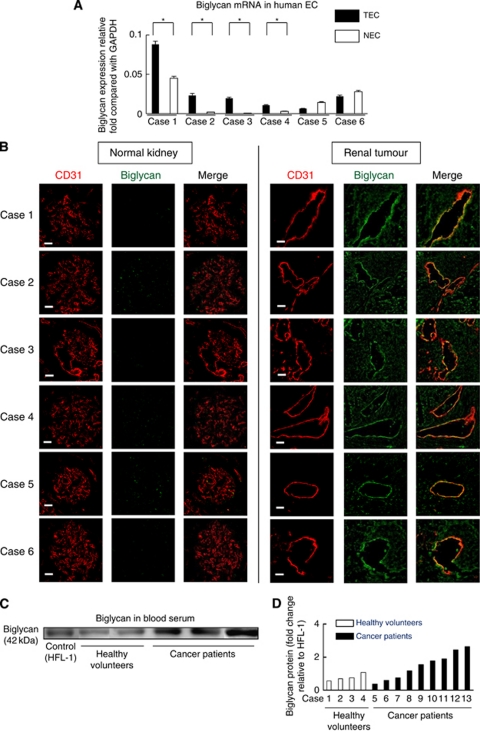
Human TECs expressed higher levels of biglycan *in vitro* and *in vivo*. (**A**) RT–PCR confirmed that biglycan was overexpressed in four of the six TEC samples compared with the corresponding NEC samples (*n*=6) (^*^P<0.05). (**B**) Tumour vessels were double stained with anti-CD31 and anti-biglycan antibodies in human renal cancer, and biglycan was expressed in tumour blood vessels but not in normal kidney vessels (*n*=6). Scale bar, 50 *μ*m. (**C**) Western blotting revealed that biglycan expression in the sera of cancer patients was upregulated compared with that in the sera of healthy volunteers. The representative data are shown. (**D**) Quantification of biglycan protein in human blood serum (*n*=13). The data are presented as fold change relative to control (Human foetal lung fibroblasts, HFL-1cell lysate 10 *μ*g).

Tumour endothelial cells and NECs were obtained from six patients. Real-time RT–PCR revealed that the biglycan expression levels were significantly higher in four of the six TEC samples than in the corresponding NEC samples (Figure 7A).

To analyse *in vivo* biglycan expression in TECs, we performed immunofluorescent double staining with anti-CD31 and anti-biglycan antibodies in the frozen sections of 11 human malignant tumours; 6 from kidneys, 3 from lungs, 1 from colon and 1 from liver. Although biglycan was hardly expressed in normal blood vessels, it was strongly expressed in tumour blood vessels (Figure 7B and Supplementary Figure S6).

To analyse whether biglycan is detected in the blood of cancer patients, glycoprotein in sera was concentrated and analysed by western blotting. Biglycan was detected in the sera from nine of cancer patients but was hardly detected in those of four healthy volunteers, and the representative results are shown (Figure 7C). The results of quantitative analysis of serum biglycan levels in each case (*n*=13) are shown (Figure 7D). Serum biglycan levels were higher in cancer patients than in healthy volunteers.

## Discussion

In this study, we detected the specific expression of biglycan in TECs isolated from xenografted tumours in mouse and human clinical cancers. We previously reported that TECs have different features than NECs in many aspects. Tumour endothelial cells exhibit a higher migratory potential than NECs (Matsuda *et al*, 2010). We, for the first time, demonstrated that biglycan, which was upregulated in TECs, might contribute to the high motility of TECs.

The retention of lipoproteins by biglycan is established as a mechanism leading to atherosclerosis (Thompson *et al*, 2011). Many of these regions have chronic inflammation. Biglycan is strongly expressed in inflammatory tissues and regulates inflammation responses via TLRs (Schaefer *et al*, 2005). We reported that TEC upregulates inflammatory molecule including COX-2 and PGE-2 (Kurosu *et al*, 2011; Muraki *et al*, 2012). These results suggest that TEC is present in chronic inflammatory environment. There seems to be a common mechanism of gene upregulation between these molecules and biglycan in TEC.

Biglycan knockdown significantly inhibited TEC migration towards VEGF, and migration was restored by exogenous biglycan treatment. Biglycan is involved in TEC migration. We have previously reported that TECs were more sensitive to VEGF with upregulation of its receptor, VEGFR (Matsuda *et al*, 2010). Because VEGFR expression did not change by biglycan knockdown (data not shown), it was suggested that biglycan knockdown inhibited TEC migratory activity independent of VEGF/VEGFR signalling. Cell migration increased in NECs after biglycan protein treatment. TLR2 and TLR4, which are reported as biglycan receptors, were expressed in TECs. Anti-TLR2 and anti-TLR4 antibodies suppressed biglycan-induced angiogenic phenotypes such as cell migration and tube formation. Because TLR2 and TLR4 are expressed on the endothelium and implicated in angiogenesis independent of VEGF, biglycan might stimulate tumour angiogenesis through TLR2 and TLR4 activation (Grote *et al*, 2010; West *et al*, 2010). Taken together, it was suggested that biglycan acts as an angiogenic factor stimulating TEC migration and tube formation in an autocrine manner through TLR2 and TLR4.

Many cancer cells such as A375SM cells, which were used to create the tumour xenografts that were the source of TECs in this study, were reported to express a low level of biglycan (Clark *et al*, 2000). Biglycan expression is either absent or undetectable in several human tumour cell lines including the epithelial carcinoma cell line A431, pancreatic adenocarcinoma cell line Miapaca2 and melanoma cell line A375SM (data not shown). We also found that biglycan mRNA was hardly expressed in human tumour stromal cells, which were negative for CD31 in a human fibroblast cell line, BJ-6, (data not shown). In *in vivo* tumour tissues, biglycan was stained in tumour blood vessels but was not or weakly stained in tumour cells and CD31-negative stromal cells including fibroblasts. It was suggested that biglycan is expressed specifically in tumour blood vessels. Furthermore, serum biglycan levels were higher in cancer patients than in healthy volunteers. These results suggested that biglycan is specifically expressed in human and mouse TECs. Biglycan secreted from TEC into blood flow might be of diagnostic value in various malignant tumours.

We analysed the effect of biglycan on vinculin, which is a key regulator of focal adhesions and participates in cell migration. Although the signalling pathway connecting biglycan and vinculin has not been elucidated, there is a report on the influence of biglycan on vinculin. Vinculin mRNA and protein expression were significantly upregulated in bgn^−/−^ fibroblasts (Melchior-Becker *et al*, 2011). We also found that TECs with biglycan knockdown were spread that was correlated with increased vinculin expression. This might be a mechanism by which cell migration was inhibited in TECs with biglycan knockdown.

For the first time, we demonstrated that biglycan might be a novel marker of TECs and is activated during tumour angiogenesis. It could be a novel target for anti-angiogenic therapy. Biglycan was highly expressed in both mouse and human TECs, and biglycan knockdown inhibited TEC migration. It might be possible to target tumour blood vessels specifically without injuring normal blood vessels using biglycan-targeted drugs in future.
