# Supplementary material for: Biglycan is a specific marker and an autocrine angiogenic factor of tumour endothelial cells
Source: Br J Cancer. 2012 Feb 28;106(6):1214–23. doi: 10.1038/bjc.2012.59 (PMC3304426; doi:10.1038/bjc.2012.59)

Yamamoto Supplemental Figure 1

The primers used for RT-PCR

NAME OF GENES	SEQUENCE
Mouse biglycan	biglycan-L:GTGTTGCTTCTTCATCTGGCTATG<br>biglycan-R: ACCTTCCGCTGCGTTACTG
Mouse GAPDH	GAPDH-L:TCTGACGTGCCGCCTGGAG<br>GAPDH-R:TCGCAGGAGACAACCTGGTC
Mouse CD31	CD31-L:TGCTCTCGAAGCCCAGTATT<br>CDC31-R:ATGGGTGCAGTTCCATTTTC
Mouse CD105	CD105-L:CTTCCAAGGACAGCCAAGAG<br>CD105-R:GGGTCATCCAGTGCTGCTAT
Mouse CD144	CD144-L: CAGCACTTCAGGCAAAAACA<br>CD144-R:TTCTGGTTTTCTGGCAGCTT
Mouse VEGFR-1	VEGFR-1-L:GAGGAGGATGAGGGTGTCTATAGGT<br>VEGFR-1-R:GTGATCAGCTCCAGGTTTGACTT
Mouse VEGFR-2	VEGFR-2-L:GGCAAATGTGTCAGCTTTGTACA<br>VEGFR-2-R:CAAAGCATTGCCCATTCGAT
Mouse CD11b	CD11b-L: GATGGGAAATGCAAAGAGGA<br>CD11b-R: AGGGTCTAAGCCAGGTCATAAG
Mosue CD45	CD45-L:CCTCAAACTTCGACGGAGAG<br>CD45-R: CACTTGCACCATCAGACACC
MouseTLR-2	TLR2-L: CGAGCTGGGTAAAGTAGAAACAGT<br>TLR4-R: AGGAGGGAATAGACAGTACTCAGGT
Mouse TLR-4	TLR4-L: GGCAGCAGGTGGAATTGTAT<br>TLR4-R: AGGATTCGAGGCTTTTCCAT
Human HB-EGF	HB-EGF-L: CGGCCGGGACCGGAAA<br>HB-EGF-R: CCTGTTTGGTGTGG
Human biglycan	biglycan-L: AGGAGGCGGTCCATAAGAAT<br>biglycan-R: AGGGTTGAAAGGCTGGAAAT
Human GAPDH	Acss3-L: ACAGTCAGCCGCATCTTCTT<br>Acss3-R:GCCCAATACGACCAAATCC

Yamamoto Supplemental Figure 2

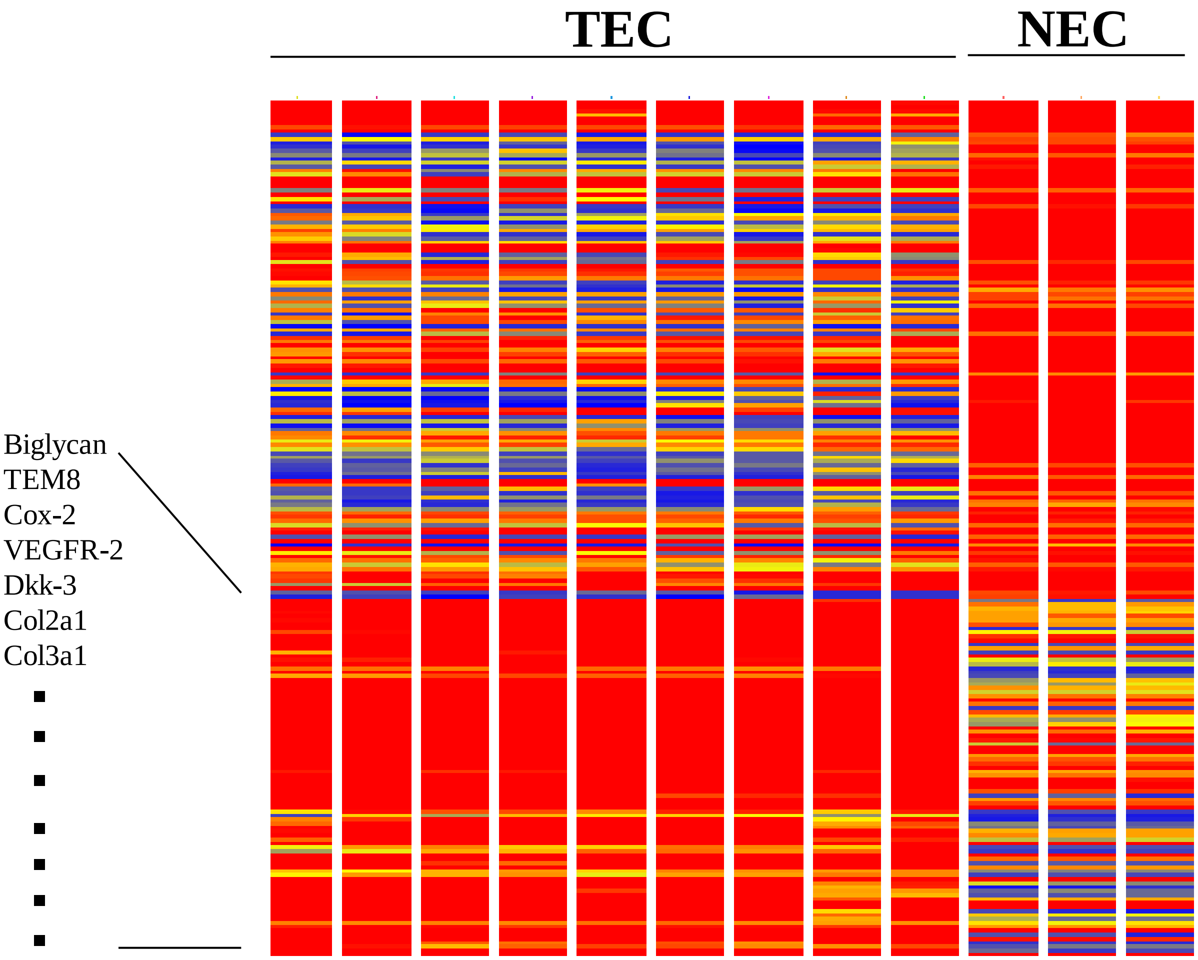

GENE	TEC>NEC (Fold Change)
Biglycan	101.96
TEM-8	11.35
COX-2	1.11
VEGFR-2	8.63
Dkk-3	261.35
Col2a1	96.70
Col3a1	3.51

Yamamoto Supplemental Figure 3

Mouse biglycan primer

forward 5′-GTGTTGCTTCTTCATCTGGCTATG-3′
reverse 5′-ACCTTCCGCTGCGTTACTG-3′

128 bp

	**Primer**	**Primability**	**stability**
Biglycan	Biglycan Fw	100%	100%
	Biglycan Rv	100%	100%
Decorin	Biglycan Fw	0%	0%
	Biglycan Rv	0%	0%
Lumican	Biglycan Fw	0%	0%
	Biglycan Rv	0%	0%
Fibromodulin	Biglycan Fw	82%	74%
※	Biglycan Rv	80%	51%

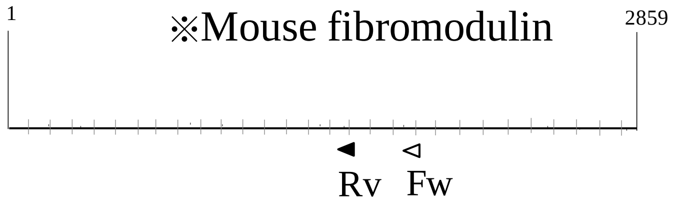

Human biglycan primer

forward 5′-AGGAGGCGGTCCATAAGAAT-3′
reverse 5′-AGGGTTGAAAGGCTGGAAAT-3′

110 bp

	**Primer**	**Primability**	**stability**
Biglycan	Biglycan Fw	85%	64%
	Biglycan Rv	100%	100%
Decorin	Biglycan Fw	83%	58%
※※	Biglycan Rv	81%	45%
Lumican	Biglycan Fw	0%	0%
※※※	Biglycan Rv		
	①	85%	64%
	②	85%	63%
Fibromodulin	Biglycan Fw	0%	0%
	Biglycan Rv	0%	0%

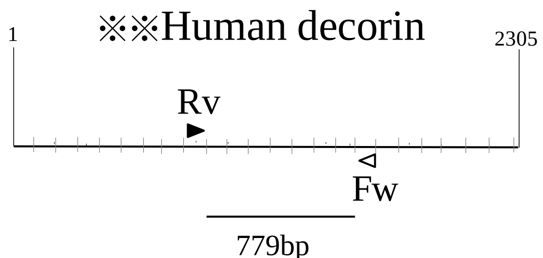

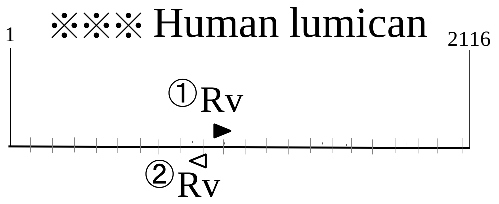

Yamamoto Supplemental Figure 4

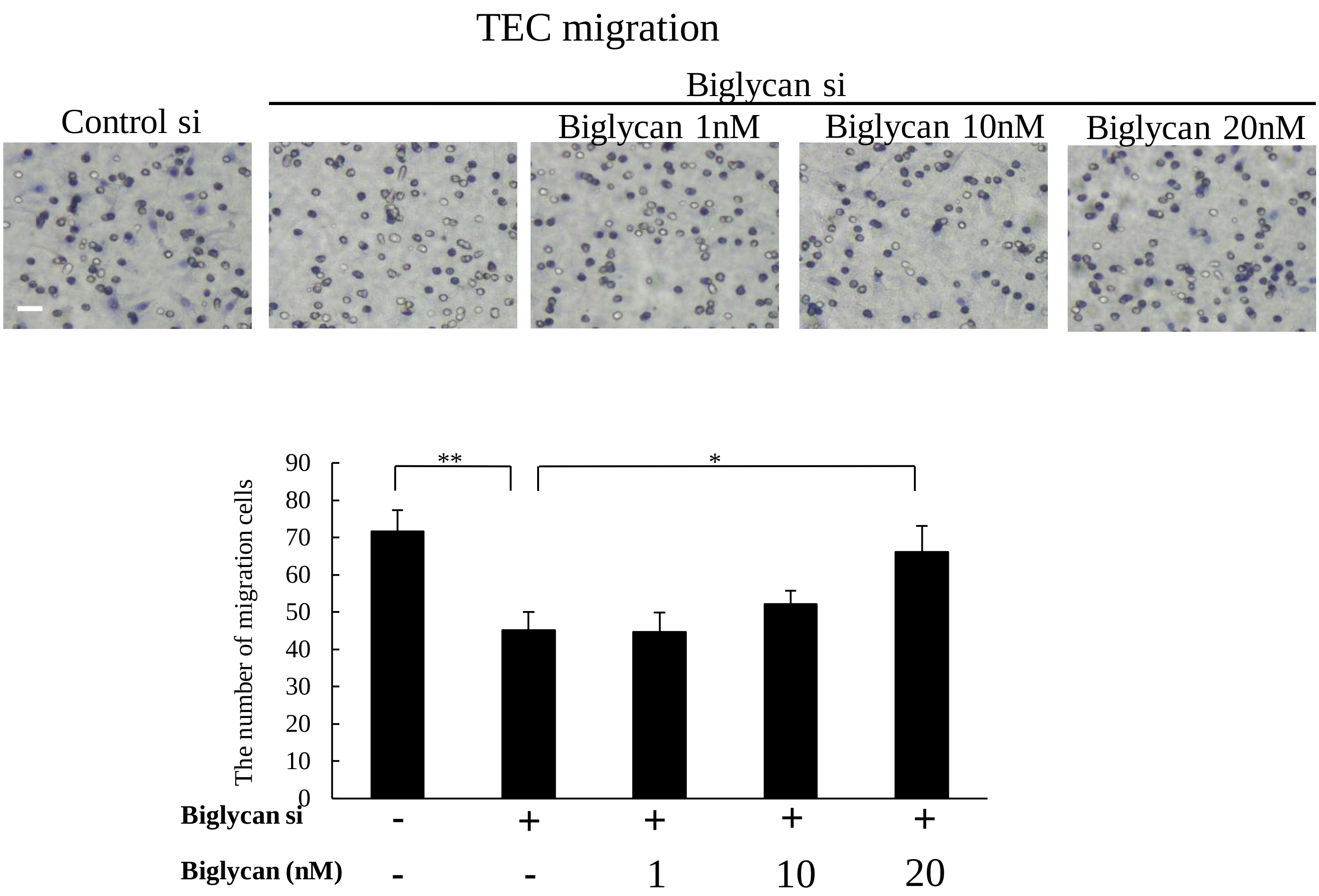

Yamamoto Supplemental Figure 5

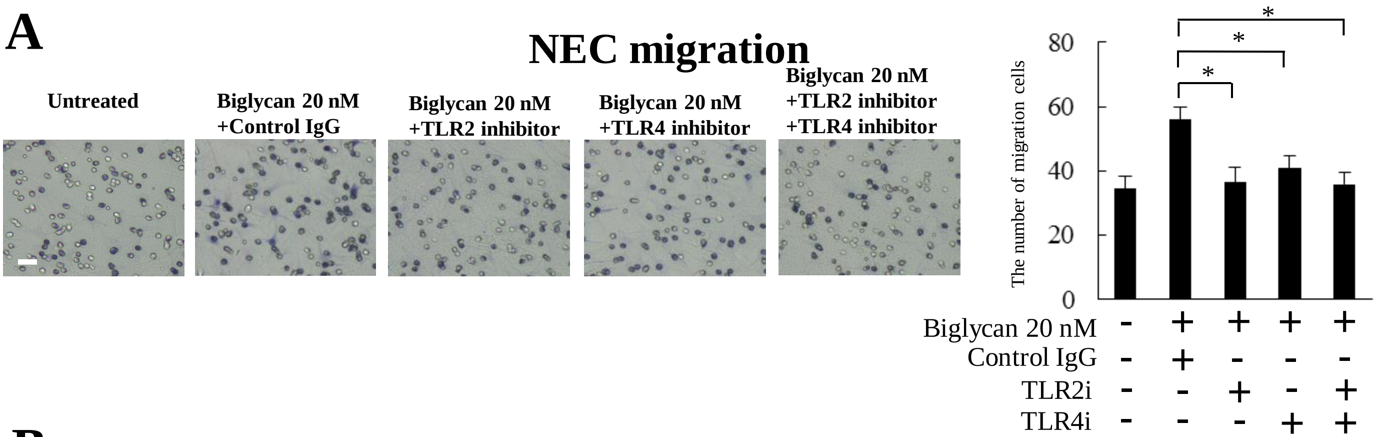

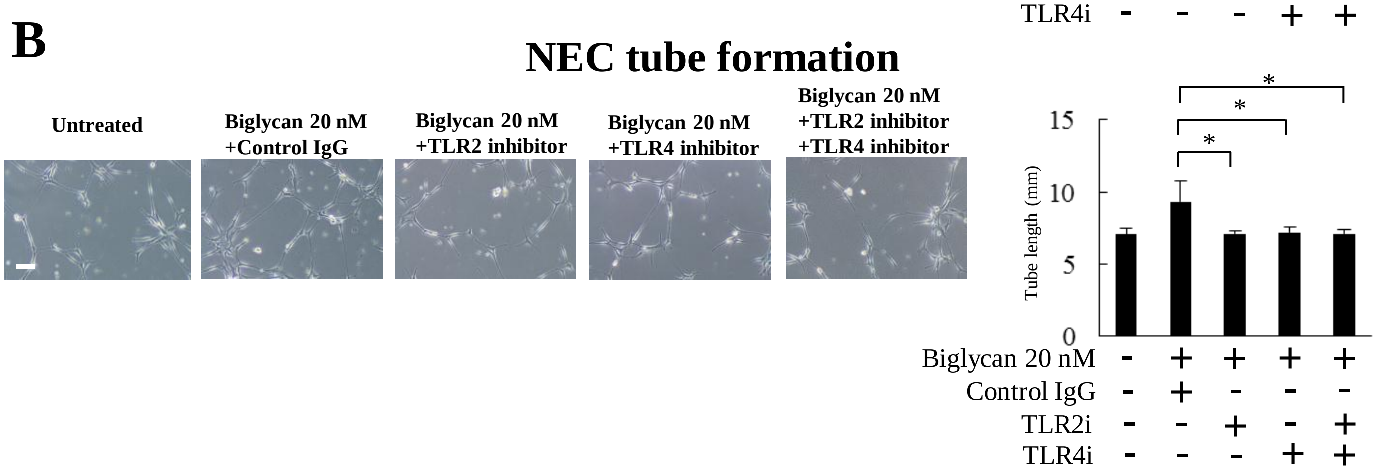

Yamamoto Supplemental Figure 6

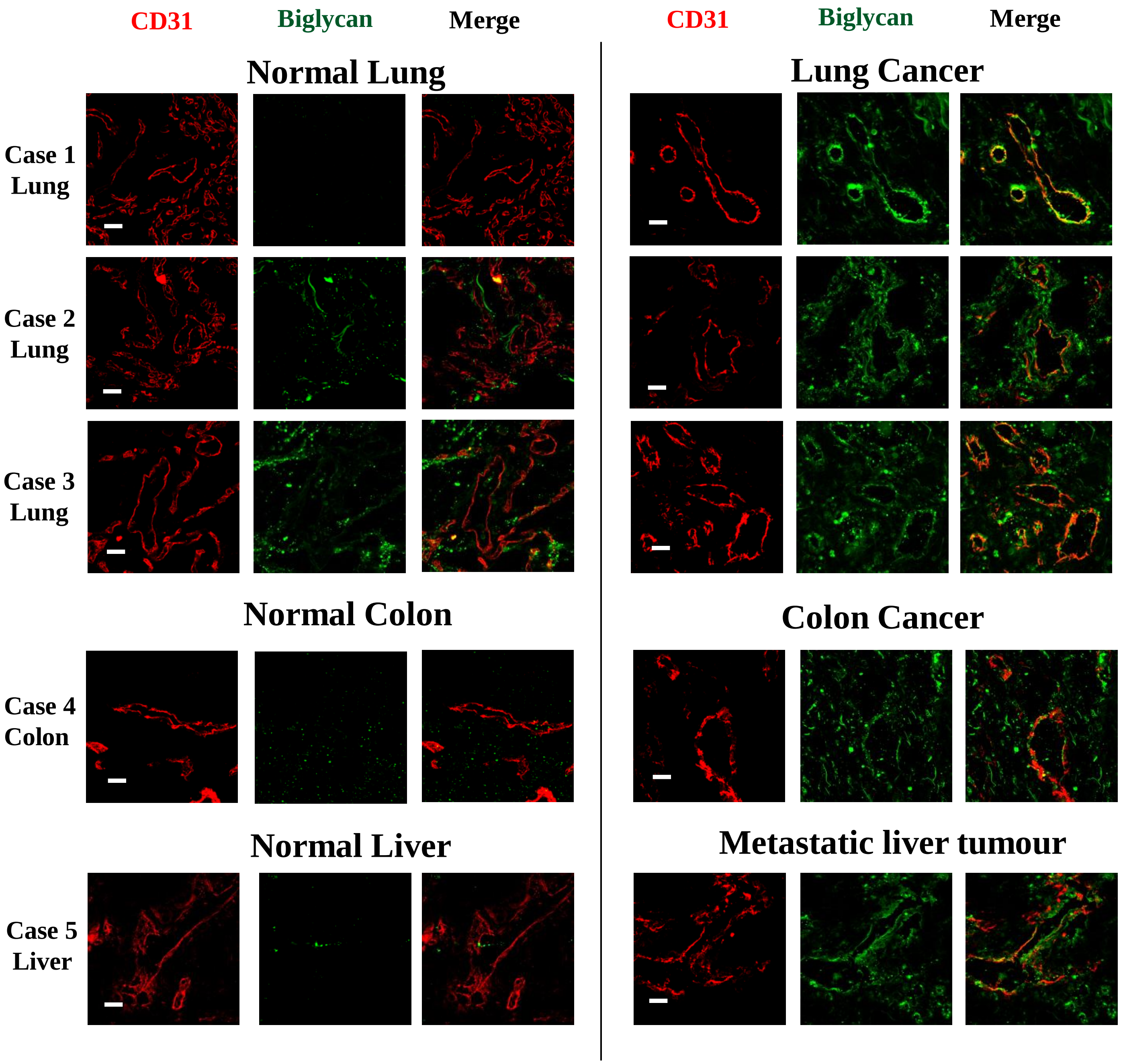

Supplement: Supplementary Figures [file bjc201259x1.pdf]
