# Supplementary material for: Biglycan is a specific marker and an autocrine angiogenic factor of tumour endothelial cells
Source: Br J Cancer. 2012 Feb 28;106(6):1214–23. doi: 10.1038/bjc.2012.59 (PMC3304426; doi:10.1038/bjc.2012.59)
Supplement: Supplementary Figure legends [file bjc201259x2.doc]

**Supplemental Figure 1**

**The Primers used for RT-PCR analysis.**

**Supplemental Figure 2**

**DNA microarray analysis of gene expression in TECs and NECs.**

The gene expression profiles of three types of TECs (melanoma-derived ECs, renal carcinoma-derived ECs and oral carcinoma-derived ECs) and NECs by DNA microarray analysis.　The gene expression levels were compared between three TECs and NEC.

**Supplemental Figure 3**

**The PCR primers for detection of biglycan.**

Oligonucleotide primers used for amplification and sequencing of biglycan gene.

Any other proteoglycans was not detected in RT-PCR. PCR product for human decorin by biglycan primer should be theoretically 779 bp, however, this size of product was not detected.

**Supplemental Figure 4**

**The analysis of the optimal concentration of the biglycan protein.**

Biglycan knockdown TECs were treated with the exogenous biglycan protein (1 nM, 10 nM, 20 nM, 40 nM and 100 nM). Cell migration towards VEGF was the most with 20 nM of biglycan. Scale bar, 100 μm.

**Supplemental Figure 5**

**Biglycan promoted cell migration and tube formation in NEC through TLR2 and TLR4.**

(A) In NEC, biglycan–induced cell migration was suppressed in the presence of blocking anti-TLR2 or anti-TLR4 antibodies. Scale bar, 100 μm

(B) In NEC, tube formation stimulated by exogenous biglycan (20 nM) was inhibited by blocking anti-TLR2 or anti-TLR4 antibodies. Scale bar, 100 μm

**Supplemental Figure 6**

**Human TECs expressed higher levels of biglycan in several tumours**

Tumour blood vessels were double stained with anti-CD31 and anti-biglycan antibodies in human lung cancer (n=3), colon cancer and metastatic liver tumours. Biglycan was expressed in tumour blood vessels but not in normal kidney vessels. Scale bar, 50 μm.
